# Effects of 90 Min Napping on Fatigue and Associated Environmental Factors among Nurses Working Long Night Shifts: A Longitudinal Observational Study

**DOI:** 10.3390/ijerph19159429

**Published:** 2022-08-01

**Authors:** Kazuhiro Watanabe, Naotaka Sugimura, Inaho Shishido, Issei Konya, Shinya Yamaguchi, Rika Yano

**Affiliations:** 1 Graduate School of Health Sciences, Hokkaido University, Sapporo 060-0812, Japan; kazu-watanabe@eis.hokudai.ac.jp (K.W.); naotaka-s@eis.hokudai.ac.jp (N.S.); ik-0v0-ik628@eis.hokudai.ac.jp (I.K.); s_yamaguchi@eis.hokudai.ac.jp (S.Y.); 2 Faculty of Health Sciences, Hokkaido University, Sapporo 060-0812, Japan; inaho_s@hs.hokudai.ac.jp; 3 Research Fellow of Japan Society for the Promotion of Science, Tokyo 102-0083, Japan

**Keywords:** environment, fatigue, long working hours, nap, nurses, shift work, sleep hygiene

## Abstract

For nurses working long night shifts, it is imperative that they have the ability to take naps to reduce fatigue, and that an appropriate environment is prepared where such naps can be taken. We verified the effects of 90 min napping on fatigue and the associated factors among nurses working 16-h night shifts. We investigated 196-night shifts among 49 nurses for one month. Wearable devices, data logging devices, and questionnaires were used to assess nap parameters, fatigue, and environmental factors such as the napping environment, ways of spending breaks, and working environment. Nurses who nap at least 90 min on most night shifts had more nursing experience. Multivariable logistic regression analysis showed that the environmental factors significantly associated with total nap duration (TND) ≥ 90 min were noise, time spent on electronic devices such as cellphones and tablets during breaks, and nap break duration. The night shifts with TND ≥ 90 min showed lower drowsiness after nap breaks and less fatigue at the end of night shift compared to those with TND < 90 min. Nurses and nursing managers should recognize the importance of napping and make adjustments to nap for at least 90 min during long night shifts.

## 1. Introduction

In nursing, shift work is vital for providing round-the-clock patient care [1,2]. In recent years, the traditional three-shift system has been replaced with a two-shift system, which entails longer working hours for nurses [3,4]. The two-shift system with long night shifts is the most common work pattern among Japanese nurses, and almost two-thirds of Japanese hospitals (64.4%) adopt night shifts of over 16 h [5]. Long night shifts are associated with intense fatigue in nurses [6,7,8]. Fatigue causes medical errors by impairing nurses’ cognitive function and performance [9,10], which can compromise patient safety [11]. Therefore, implementing countermeasures to reduce fatigue is important to patient safety when nurses engage in long night shifts.

The greatest cause of fatigue is insufficient or disrupted sleep [12]. Napping is a countermeasure to fatigue recommended by the American Nurses Association and Japanese Nursing Association [13,14]. Although systematic reviews and meta-analyses of napping during night shifts have also shown that taking naps can reduce fatigue, the optimal duration and timing have not been identified [15,16,17]. Nap duration ranging from 15 to 180 min has been widely reported for nurses [15], but it is important to note that nap breaks and sleep duration are discussed together as nap duration. This is because some previous studies have focused on the implementation of napping and ensuring the duration of nap breaks during night shifts [18,19]. For example, although Smith-Coggins et al. examined the effects of a 40-min nap on fatigue [19], this length represented only nap break duration rather than total sleep duration. Moreover, sleep duration can be further divided into subjective aspects such as maintaining a sleep diary [18,20], and objective aspects such as an actigraphy [21,22]. We must distinguish between these durations to establish quantitative evidence without discussing the effects of napping by comparing the nap vs no-nap groups alone. Moreover, to measure the outcomes of effects on napping during night shifts, the outcomes must be integrated across multiple time points. This is considered the effect of sleep inertia, defined as the brief period of decreased cognitive function or performance immediately after a period of sleep that can temporarily obscure the recuperative effects of sleep [23]. In a study that investigated 196 night shifts of 16 nurses, the nap group showed higher sleepiness, one sign of fatigue, at 4:00, 5:00 and 6:00 than did the no-nap group; however, sleepiness was reversed at the end of the night shifts, so the nap group had lower sleepiness [20]. In other words, evaluating only a single point outcome during the night shift is insufficient. Furthermore, the literature has provided minimal justification for selected nap durations [17]. The Japanese Nursing Association suggests that two-hour nap breaks are required for nurses to be able to take a 90 min nap [14], which corresponds with the duration of one non-rapid eye movement (NREM)/rapid eye movement sleep cycle based on sleep physiology [24]. Slow wave sleep, the deepest of NREM, is considered to play an important role in energy recovery [25]. However, since the effects of 90 min napping during night shifts on fatigue among nurses have not been identified, we must measure napping quantitatively and measure fatigue at multiple time points.

In addition, the environments in which nurses nap are inadequate [18,26,27]. Environments refer to sleep hygiene [28] and the working environment. Nurses’ napping places are reported to be their restrooms with mattresses placed in an isolated section [29,30], storage rooms with reclining armchairs [20], stretchers, and bedsheets on the floor [31]. However, the environmental factors related to sleep, such as light, noise, temperature, and humidity [32,33,34], have not been investigated in nurses’ napping places. Sleep hygiene behaviors, such as caffeine intake [28], the use of electronic devices such as cellphones and tablets [35], and diet [36], may also be associated with napping during night shifts. However, there are no reports on how nurses spend their nap breaks aside from napping. Additionally, the working environment is another important factor associated with nurses’ napping. One review identified a high prevalence of missed, interrupted, or delayed rest breaks due to job demands and resources [37]. Despite these factors, the relationship between the environment and napping during night shifts has not been verified [23].

Conducting field studies is also important to expand the evidence of the effects of napping during night shifts on fatigue [17]. Nurses’ working environments, including nap breaks, differ day-to-day depending on job demands [20,31]. Hence, a longitudinal study including these environmental factors should be conducted. We aimed to verify the effects of 90 min napping on fatigue and the associated factors among nurses working 16-h night shifts. Initially, we explored which nurses were able to nap at least 90 min during four night shifts. We also explored factors related to taking a 90 min nap in terms of the environmental factors such as napping environment, ways of spending breaks, and working environment. Finally, we verified the effects of 90 min napping during night shifts on reducing fatigue. These findings could serve not only as evidence for a better environment for napping and developing effective fatigue countermeasures for nurses, but also as data for nurses’ managers and researchers to consider while developing strategies to tackle these issues.

## 2. Materials and Methods

### 2.1. Operational Definition

Napping: A nap is a short duration of sleep taken during a night shift. In this study, total nap duration (TND) was calculated using a wearable device.

### 2.2. Study Design

The longitudinal investigations of night shifts were conducted over the course of one month for each nurse. As the average number of night shifts per month for nurses in Japan is more than four to less than five [38], we investigated four night shifts per nurse between July and September 2019. To control the working environment, the investigation included only one facility that had adopted night shifts of over 16 h.

### 2.3. Participants

The participants comprised nurses from six wards in a general hospital with more than 200 beds located in northern Japan. The quantity and quality of sleep differ with age, for example, one of the reasons for this is hormonal imbalance associated with menopause occurring in women who are in their 40s [39,40,41]. Therefore, we recruited nurses aged 20–39 years to control for age-related factors as a priority in this study. The exclusion criteria were as follows: first year of clinical experience; regular use of sleeping pills; diseases under treatment; and pregnancy. The sample size was determined by nurses who fulfilled the criteria. Of the 55 nurses in the hospital who were eligible, 50 consented to participate. Ultimately, 49 nurses from all six wards were analyzed (one nurse withdrew from the study). Table 1 shows the participants’ basic attributes.

### 2.4. Work Condition

The hospital adopted a two-shift system with a night shift of over 16 h (from 16:30 to 9:00 the following day). Nurses are permitted to have both a supper break and at least two hours of nap break during the night shift. Nurses take a nap break after discussing the order of nap breaks among staff during the night shift. Nap break duration was basically evenly distributed among the staff, except when an event occurred.

### 2.5. Measures

#### 2.5.1. Nurses’ Characteristics

Demographic data on age, years of nursing experience, years of nursing experience in the current ward, sex, marital status, and child-rearing were obtained as described above. Regarding usual sleep, we asked about the subjective mean daily sleep duration and whether or not they usually take a nap before the night shift. A self-assessment version of the Morningness–Eveningness Questionnaire [42,43], including 19 multiple-choice questions regarding sleep habits and preferences with a total score ranging from 16 to 86, was used to assess chronotype. The nurses were classified into five categories based on the total score: “definite morning type (total score: 70–86),” “moderate morning type (59–69),” “intermediate type (42–58),” “moderate evening type (31–41),” and “definite evening type (16–30).” Data on sleep-related habits were obtained using a questionnaire comprising frequency of caffeine intake, daily time spent on electronic devices (e.g., cellular phone, smartphone, tablet) during the day and before bedtime.

#### 2.5.2. Sleep/Activity

The MTN-220 (ACOS CO., LTD., Iida, Japan) was used to assess sleep/activity. The reliability of this device has been verified based on polysomnography [44]. This device (diameter, 27 mm; thickness, 9.1 mm; weight, 9 g) records the amount of activity using an internal three-axis accelerometer. Posture can also be determined from six directions. The nurses were required to clip it to their uniform on the front side of the trunk only during the night shift. The amount of activity and posture were recorded every two mins.

Data were analyzed using the Sleep Sign Act ver.2.0 (SSA) software (KISSEI COMTEC CO., LTD., Matsumoto, Japan). To detect sleeping and awaking duration from the MTN-220 data, the in-bed and out-of-bed times were manually determined with reference to the subjective napping time reported by the nurses. The default settings in the SSA were used, with the parameters detected following a previously reported algorithm [45].

Optimizing the definitions of napping parameters:

1. TIB (min): Time in bed. Duration with a lying posture.
2. SL (min): Sleep latency. The interval duration between the time of changing posture from standing to lying and the first sleep-onset time.
3. WASO (min): Wake after sleep onset. The total duration one stays awake during the sleep onset to sleep offset interval.
4. BOT (min): Bed-out time. The interval duration between the last awakening time and the time of changing posture from lying to standing.
5. TND (min): Total nap duration. The TIB from which SL, WASO, and BOT were subtracted; sleeping duration.
6. SE (%): Sleep efficiency. The ratio of TND to TIB. This represented napping quality.

To calculate the awakening duration until nap breaks as an indicator of the homeostatic sleep pressure, the last sleep duration and awaking time before the night shift were recorded using a questionnaire. For the subjective evaluation of quality, the satisfaction level of napping was assessed using a questionnaire with the usual main sleep as 100%.

Steps between 17:00 and 9:00 the following day were calculated using the same software as an index reflecting workload [46]. The steps for each night shift were divided into two categories: steps before and after napping. The steps before napping were calculated as the steps taken every hour from 17:00 to time spent in bed, while the steps after napping were from time spent outside bed to 9:00.

#### 2.5.3. Napping Environment

We measured illuminance, temperature, humidity, noise, unpleasant smells, bedding, and the napping places as a napping environment. Illuminance, temperature, humidity, and noise were defined as the mean values corresponding to the napping (from the start to end time of the napping) or time in bed if the nurses were unable to nap detected by the wearable device during each night shift. In order to measure illuminance, temperature, humidity, and noise, we prepared data logger devices on a wagon (65 cm above the floor) for the number of nurses working the night shift. Nurses were required to carry them to their napping place and place them as close to their heads as possible during their nap breaks.

Light levels in napping places were measured using a wireless thermo-hygrometric illuminance meter (LogMiru^®^ BT HLT-100BT, CUSTOM Corporation, Tokyo, Japan). This sensor includes an environmental light integrated circuit, which measures light intensity with a range of 0–30,000 lx with a resolution of 1 lx and an accuracy of ±20% reading. In this study, measurements were recorded every minute and stored in the data logger’s internal memory. Temperature and humidity were measured using the same device as the illuminance meter (LogMiru^®^ BT HLT-100BT, CUSTOM). The sensor included a thermistor measuring temperature within a range of −10–50 °C with a resolution of 0.1 °C and an accuracy of ±1 °C. The ceramic resistance humidity sensor had a range of 20–95% relative humidity, a resolution of 1%, and an accuracy of ±5%. Noise was measured using a digital sound level meter with a data logger (GM1356, Suzhou SUTEKI Information Technology Co., LTD, Suzhou, Jiangsu Province, China) for every 15 sec period during night shifts. Microphones were unified in an upward direction and placed on the wagon. Based on the above data of fast, A-weighted sound level, equivalent sound level (dB *L*_Aeq_) over a given period of time (napping) was calculated as noise level. Unpleasant smells were judged subjectively and recorded after the night shift. Details regarding bedding and napping places were also asked in the original questionnaire.

#### 2.5.4. Ways of Spending Breaks

At the end of the night shift, the original questionnaire was used for the nurses to describe how they spent their nap breaks, including naps. To consider the lasting effects of caffeine, caffeine consumption before nap breaks was checked. The questionnaire also asked about the amount of time spent using electronic devices. Additionally, the subjective time of falling asleep, subjective time of awakening, carrying a personal handy-phone system (PHS) with the nurse call system, changing clothes, using a sleep mask, using earplugs, watching television, listening to music, doing remaining work, and eating were also confirmed.

#### 2.5.5. Working Environment

After the night shift, the nurses were asked about the working environment during the night shift using the original questionnaire. Data regarding the ward, including the number of nurses, care workers, hospitalized patients, and patients each nurse was responsible for, and nap break duration (discussed among staff during night shift), were collected. As well as data regarding individuals, such as supper breaks and the start and end times of nap breaks, were also obtained.

#### 2.5.6. Fatigue

Based on previous study [21], the following two questionnaires are used to assess work-related fatigue: Cumulative fatigue was measured using the Cumulative Fatigue Symptoms Index (CFSI) [47]. The CFSI comprises 81 yes/no questions in eight categories: “general fatigue,” “chronic fatigue sign,” and “physical disorders” for physical aspects; “depressive feelings,” “anxiety,” and “decreased vitality” for mental aspects; and “irritability” and “unwillingness to work” for social aspects. The complaint rate was calculated as follows:

Complaint rate in each category (%) = (number of positive items in each category/[number of items in each category × number of participants]) × 100.

The nurses were asked to complete the CFSI at the beginning of the fourth night shift.

Feelings of fatigue during night shifts were measured using the *Jikaku-sho shirabe* questionnaire developed by the Research Group of Industrial Fatigue, a part of the Japan Society for Occupational Health [48]. This questionnaire includes 25 subjective fatigue symptoms categorized into five factors: (i) drowsiness; (ii) instability; (iii) uneasiness; (iv) local pain or dullness; and (v) eyestrain. All items are based on a 5-point rating scale (1 = *disagree at all* to 5 = *agree strongly*). The total score of each factor and the overall total score were used. The nurses completed the questionnaire four times per shift: at the beginning of the night shift; before a nap break; after a nap break; and at the end of the night shift.

### 2.6. Statistical Analysis

Descriptive statistics were computed for each variable. Continuous variables were presented as means and standard deviations or medians and interquartile ranges, while categorical variables were described as frequencies and percentages. Since the data in this study were measured four times, there is a data hierarchy based on the nurses (Level-2) and the night shift (Level-1). Intra-class correlations (ICC) were checked during the analysis.

First, to explore the characteristics of at least 90 min napping, 196-night shifts of Level-1 for the study period were divided into napping with TND ≥ 90 min and TND < 90 min groups using the calculated TND values. When the two values of TND ≥ 90 min and TND < 90 min group were used as dependent variables, the ICC was close to zero (<0.001) under the assumption that the logistic distribution [49]. So, we assumed the TND group to be independent between night shifts. We explored the factors associated with TND ≥ 90 min separately for nurse-dependent (Level-2) and night shift-dependent (Level-1) variables, respectively.

Regarding the nurse-dependent (Level-2) variables, we classified nurses into five groups (none, one, two, three, and four night shifts) according to the number of times they were able to take a nap of at least 90 min during four night shifts. Kruskal-Wallis and Fisher’s exact tests were conducted to compare the characteristics and cumulative fatigue among these groups. Post hoc tests were conducted using the Steel-Dwass test.

In terms of night shift-dependent (Level-1) variables, the napping parameters of the TND ≥ 90 min and TND < 90 min groups were compared using student’s *t*-tests. To identify the environmental factors associated with TND ≥ 90 min, student’s *t*-tests or Fisher’s exact tests were performed for each of the napping environments, ways of spending breaks, and working environments between groups. Variables with *p* < 0.20 were included in a multivariable logistic regression analysis to identify significantly associated factors. Adjusted odds ratios (a*OR*s) and 95% confidence intervals (CIs) were then calculated. Awakening duration until nap breaks, the interval from the last awakening time before the night shift to the start time of nap breaks, was used as a covariate to adjust the homeostatic sleep pressure [50,51].

To examine the effect of 90 min napping on fatigue during night shifts, mixed linear models were used based on a previous study [20]. The ICCs of all feelings of fatigue from nurses were from 0.08 to 0.15. The measures that were repeated during the night and on different nights during the study were included in the model as two random hierarchical effects, to take into account the correlation between these measures. One random effect was added for the night shift and another random effect for the nurses. To compare changes in each feeling of fatigue between the TND groups, we added TND groups, time (before nap breaks, after nap breaks, and at the end of the night shift), and their interactions as a fixed effect. Furthermore, to compare feelings of fatigue between the groups each measure point, post hoc *t*-tests were conducted using estimates of mixed linear models.

All other analyses were conducted using JMP^®^ Pro software, version 16.0 (SAS Institute Inc., Cary, NC, USA) and R version 4.2.0 [52], and statistical significance was set at α = 0.05.

## 3. Results

### 3.1. Nurses’ Characteristics

The characteristics of the nurses are shown in Table 2, excluding basic attributes. The subjective mean daily main sleep duration was 6.3 h, and the most common chronotype was intermediate. The most common duration for the daily use of electronic devices was more than 2 h (61.2%) and that before bedtime was between 30 and 44 min (34.7%).

### 3.2. Characteristics and Cumulative Fatigue of Nurses Based on the Number of Naps with TND ≥ 90 Min during Four Night Shifts

**Figure 1:**
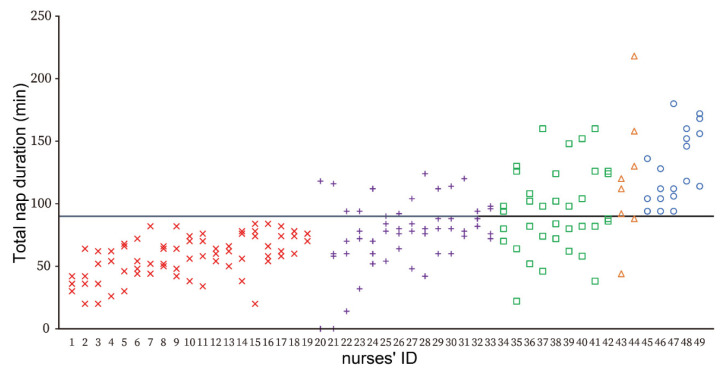
The distribution of total nap duration for each nurse’s during four night shifts. **Notes.** The vertical axis indicates total nap duration. The horizontal axis indicates the nurses’ ID. The auxiliary line show that the total nap duration is 90 min. Nurses were classified into five groups according to the number of times they were able to take a nap of at least 90 min during their four night shifts. Red crosses: None group (*n* = 19), Purple pluses: One night shift group (*n* = 13), Green squares: Two night shifts group (*n* = 10), Orange Triangles: Three night shifts group (*n* = 2), Blue circles: Four night shifts group (*n* = 5), respectively. **Abbreviations.** ID, identification.

The distribution of TND for each nurse’s four night shifts is shown in Figure 1. When all nurses were categorized by the number of naps with TND ≥ 90 min, 10.2% napped for TND ≥ 90 min all four night shifts (*n* = 5), while the proportions of the nurses who napped during three, two, and one night shift(s) were 4.1% (*n* = 2), 20.4% (*n* = 10), and 26.5% (*n* = 13), respectively. In contrast, 38.8% were unable to nap for at least 90 min during any of their night shifts (*n* = 19). Because few nurses napped during three night shifts, they were reclassified into three groups (none, one/two night shifts, and three/four night shifts). The three/four night shifts group had significantly more years of clinical experience than the none group (Table 3).

Next, we compared the cumulative fatigue in three nurses’ groups. The results showed no significant difference in CFSI (Figure S1).

### 3.3. Characteristics of Napping with TND ≥ 90 Min

During all 196-night shifts analyzed, the nurses managed to take breaks to lie down. The number of night shifts with TND ≥ 90 min was 59 (30.1%) compared with 137 for those with TND < 90 min (69.9%). Table 4 lists the napping parameters for both groups. Compared with the TND < 90 min group, the TND ≥ 90 min group showed significantly longer TIB, shorter SL, higher SE, and a later end time of napping. The TND ≥ 90 min group also had a longer WASO and a higher frequency of awakenings and postural changes. Subjective evaluation was significantly higher in the TND ≥ 90 min group. There was no significant difference in the start time of napping and awakening duration until nap breaks between the two groups.

### 3.4. Napping Environment, Ways of Spending Breaks, and Working Environment during Night Shifts

Table 5 lists the napping environment, ways of spending breaks, and working environments in both groups. For the napping environment, the nap area (restrooms, conference rooms, informed consent rooms, and examination rooms) was fixed in each ward; however, some nurses used beds in vacant hospital rooms. Bedding was also fixed in the nap area. For most of the nurses, the average illuminance during napping was less than 10 lx; in contrast, only one nurse had an average of more than 500 lx on all night shifts. The lighting equipment comprised either a downlight with low color temperature (warm color) or a fluorescent light with high color temperature (cold color); none of these were LEDs. Noise level in the TND ≥ 90 min group was significantly lower than that in the TND < 90 min group (*p* < 0.001). In this study, the higher noise level was not related to the longer WASO, higher frequency of awakenings, and postural changes (Figure S2). In contrast, there were no significant differences in temperature and humidity.

Regarding the ways of spending breaks, excluding napping, the use of electronic devices was the most common (mean use = 27.9 min). The reasons for use were listed as setting alarms, using social networking sites, watching videos, and using the Internet. In 48 night shifts (24.5%), the nurses ate something in addition to supper; most of these were light meals such as rice balls, bread, and sweets. Although the scheduled nap breaks were not interrupted, some completed the remaining work at their own discretion, even during nap breaks.

For the working environment, all of the nurses could take nap breaks for over 90 min. The nap break duration of the TND ≥ 90 min group was significantly longer than the TND < 90 min group (*p* < 0.001). For 24 night shifts, a part of the activity data could not be recorded due to a bug. Hence, we calculated the steps excluding the bug part. Even under these conditions, the number of steps before napping per hour of the TND ≥ 90 min group was significantly lower than the TND < 90 min group (*p* = 0.014).

### 3.5. Environmental Factors Associated with a Night Shift of TND ≥ 90 Min

The univariate analysis showed that the factors associated with TND ≥ 90 min were noise level, humidity, Japanese futons, folding beds, stretchers, examination tables, use of toweling blankets, carrying a PHS with the nurse call system, time spent on electronic devices, doing remaining work, eating, number of care workers, number of hospitalized patients, start time of nap breaks, nap break duration, and steps before napping per hour (Table 5). Multivariable logistic regression analysis was performed using these variables. Stretchers were excluded from this analysis due to making the model unstable (no night shifts using stretchers corresponded to TND ≥ 90 min). As all the variance inflation factors were below three, there were no signs of multicollinearity. The factors between night shifts significantly related to TND ≥ 90 min were noise level from the napping environment (a*OR*: 0.88 [95% CIs: 0.78, 0.98]), time spent on electronic devices from the ways of spending breaks (0.97 [0.94, 0.99]), and nap break duration from the working environment (1.04 [1.02, 1.07]) (Table 6).

### 3.6. Relationship between Naps with TND ≥ 90 Min and Feelings of Fatigue

**Figure 2:**
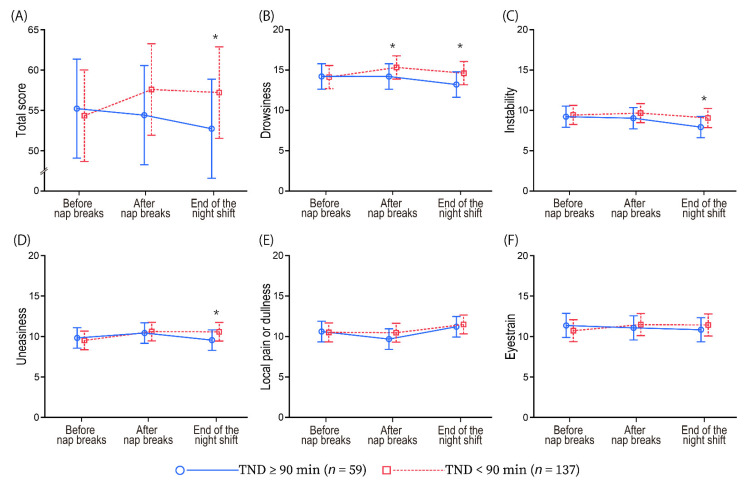
Comparison of change in feelings of fatigue between night shifts with TND ≥ 90 min (*n* = 59) and TND < 90 min (*n* = 137) groups. **Notes**. The sample units in this figure are the number of night shifts. The vertical axis indicates the least squares means in feelings of fatigue (*Jikaku-sho shirabe*), with higher scores showing higher fatigue. The horizontal axis indicates the measurement points. The blue line is TND ≥ 90 min group, while the red line is TND < 90 min group. Error bars represent 95% confidence interval. (**A**): total score, (**B**): drowsiness, (**C**): instability, (**D**): uneasiness, (**E**): local pain or dullness, (**F**): eyestrain, respectively. The least squares means were analyzed using the mixed linear model, while post hoc *t*-tests were conducted using their estimates to compare between groups each measurement point. **Abbreviations.** TND, total nap duration. * *p* < 0.05.

The mixed linear models showed that significant interactions (TND group × times) were observed for drowsiness (*F* [2, 387.7] = 4.35, *p* = 0.013), uneasiness (*F* [2, 387.5] = 7.15, *p* < 0.001), local pain or dullness (*F* [2, 387.4] = 3.29, *p* = 0.038), eyestrain (*F* [2, 387.6] = 4.36, *p* = 0.013), and total score (*F* [2, 387.5] = 6.90, *p* = 0.001), except instability (*F* [2, 387.5] = 2.98, *p* = 0.052). Post hoc tests showed that the drowsiness of the TND ≥ 90 min was significantly lower than that of the TND < 90 min at the after nap breaks. At the end of night shifts, the drowsiness, instability, uneasiness, and total score of TND ≥ 90 min were significantly lower. On the contrary, all scores before nap breaks were not significantly different. Figure 2 shows the fatigue change and comparison after nap breaks and at the end of the night shift between the TND groups based on the least square means.

## 4. Discussion

### 4.1. Napping with TND ≥ 90 Min and Associated Factors

Longitudinally and repeatedly investigating the nurses’ night shifts for a month, we verified the factors associated with 90 min napping both in terms of nurses’ characteristics and the environment during the night shift.

Regarding napping during the night shift, although the nap breaks for all nurses were longer than 90 min, only 59 night shifts (30.1%) had TND ≥ 90 min. Only five nurses took naps of at least 90 min on all four night shifts. These results show the difficulty in sleeping long during working hours, even at night. There was no significant difference between the groups for the awakening duration until nap breaks, start time of napping, and drowsiness before nap breaks, suggesting that the homeostatic sleep pressure before the nap was probably at the same level in this study. In other words, other factors may affect TND.

One of the characteristics of nurses who slept for TND ≥ 90 min during most night shifts was more years of nursing experience, even when they were aged 20–39 years. This result is probably “the healthy shift worker effect,” which has been reported to be related to age and other individual attributes [53]. This effect has been explained as older workers representing a selection of workers who may be healthier, can cope well with shift work, and wish to stay in shift work [54]. The results of this study suggest that nurses are able to take enough naps every night shift, which may be one of the factors contributing to this effect. However, we are unable to conclude it in this study, since only five nurses slept TND ≥ 90 min on all night shifts. On the other hand, based on the distribution of TND in this study, it was difficult to explain TND ≥ 90 min by individual factors alone.

We focused on night shift-dependent variables, which suggested that TND ≥ 90 min was associated with three environmental factors: noise, time spent on electronic devices during nap breaks, and nap break duration. Noise was associated with TND ≥ 90 min, which is with the fact that many aircraft maintenance engineers report noise as preventing them from napping during night shifts [55]. Hence, noise may be a common issue when napping in the workplace. Hospitals are prone to noisiness, which comes from diverse noise sources such as conversations, the operation of medical equipment, and the coughing and snoring of patients [56]. In this study, each napping place was located in a public room used for other purposes. Therefore, since no positive correlation was found between noise level and indicators of body movement such as, WASO, the frequency of awakenings, and postural changes, those noise levels were considered to be more reflective of the napping place than the nurses’ self-generated sound. According to the Night Noise Guidelines for Europe, if negative effects on sleep are to be avoided, the equivalent sound pressure level should not exceed 30 dBA indoors for continuous noise [57]. Although both TND groups in this study exceeded this criterion, the noise level 39.6 dB *L*_Aeq_ of TND ≥ 90 min was lower than that of TND < 90 min. The results objectively support that nurses desire quiet nap rooms [26], and they may nap longer in such rooms. Providing nurses with a quiet room will be beneficial to allot time for effective naps during their shifts. Incidentally, we found no evidence that illuminance was associated with nurses’ napping. Light is an important factor that synchronizes circadian rhythms and influences sleep. Another nap room attribute desired by nurses is low lighting [26], because light exposure at night suppresses melatonin and reduces sleepiness [58,59]. Because the nurses in this study could control the lighting in the napping places, most set up an appropriate environment for themselves by turning off the lights during napping. Similarly, we found no evidence that temperature and humidity were associated with napping because they were properly maintained by the air conditioning in the hospital. Hence, we cannot conclude that illumination, temperature, or humidity did not affect napping based on the results of this study.

Regarding ways of spending breaks, time spent on electronic devices was related to TND ≥ 90 min. The use of electronic devices in the TND ≥ 90 min group was relatively short. The use of electronic devices during limited nap breaks reduces nap duration. To nap for a sufficient duration, time spent on electronic devices should be as short as possible. In this study, we could not determine the timing of the use of electronic devices. In the TND ≥ 90 min group, the mean time spent on electronic devices was 23.6 min, while in the TND < 90 min group it was 29.8 min. We confirmed that the mean interval time between the last awakening time of napping and the end time of nap breaks for the TND ≥ 90 min group was 15 min, while for the TND < 90 min group it was 14 min. Therefore, we assume that most of them used mobile devices before napping. In addition, as most of the nurses answered that they always use their electronic devices before bedtime daily, they may use them before napping as well. The use of light-emitting electronic devices before bedtime prolongs the time it takes to fall asleep and increases alertness, even in low-light conditions [32,60]. In addition, sleep disorders such as insomnia can be induced by the increased use of electronic devices at bedtime [61,62], which may have affected nurses’ napping during night shifts. However, considering that the nurses themselves chose to spend time on electronic devices, the decision regarding their use should be discussed after a multifaceted evaluation of their psychological and other effects. Therefore, the relationship between electronic device use and napping during the night shift requires further investigation, including time and timing of use as well as content viewed on the device.

Nap break duration was a related factor in the working environment. Even the TND ≥ 90 min group, which had better sleep quality than the TND < 90 min group, took an average of 17.2 min to fall asleep. If the napping place is not located in a room dedicated only to napping, bedding is necessary to nap, and it takes time to prepare. Thus, to take at least a 90 min nap, a sufficient nap break duration is required. However, staffing levels are lower during night shifts [18,27], making it difficult for nurses to improve this situation. Nursing managers must be aware of these issues and consider countermeasures.

When the factors for both nurses and night shift are considered together, nurses with longer nursing experience may be better able to manage the environment.

### 4.2. Effects of TND ≥ 90 Min on Fatigue

Our study showed that the changes in fatigue between the TND ≥ 90 min and TND < 90 min groups were different, as shown in Figure 2. Night shift nurses who were able to nap for at least 90 min were less drowsy after nap breaks and less fatigued at the end of the night shift compared with the TND < 90 min group.

Regarding after nap breaks, previous studies have reported that sleep inertia is likely to occur after nighttime napping, and nurses require attention [19,63]. Sleep inertia normally lasts about 30 min, but napping during the night shift may exacerbate the effects in terms of circadian rhythm [64]. In addition, while previous studies support short naps during the night shift [65,66], there is little evidence to support long naps. Nonetheless, it is interesting that the TND ≥ 90 min group had lower drowsiness after napping in this study. Moreover, the study contrasted with those of Takahashi et al., who reported that post-nap fatigue was higher with an increased nap length in nurses who were allowed two-hour nap breaks [67]. The reason may be that the previous study discussed the TIB, which was determined from the posture as well as the nap length, whereas we validated it with the nap duration. In the previous study, TIB was longer, but TND could have been shorter. Therefore, sleep efficiency may have been lower. When the same analysis as in the previous study was performed in this study, there was no correlation between TIB and fatigue (Figure S3), but a negative correlation was observed with TND (Figure S3). Thus, the results also suggest that the TND, actual sleep duration, is more important than TIB to discuss reducing fatigue.

At the end of the night shift, the result that the TND ≥ 90 min group showed lower fatigue concurred with a previous study that found less fatigue in the morning [19,20]. The reason for the lower fatigue was that naps of at least 90 min had good quality as well as quantity. The TND ≥ 90 min group fell asleep more easily and had higher sleep efficiency and subjective satisfaction than the TND < 90 min group. Moreover, drowsiness in the TND ≥ 90 min group was also lower at both time points. The highest level of sleepiness is recorded in the early morning hours for most individuals [68]. Napping for at least 90 min may also be effective at reducing or suppressing sleepiness in the morning. Higher fatigue and sleepiness cause cognitive impairment and performance deficits [9,10]. Thus, napping for at least 90 min may help prevent accidents and ensure patient safety [69].

On the other hand, there was no significant difference in the cumulative fatigue be-tween three nurses’ groups categorized by the number of TND ≥ 90 min during four night shifts. The result suggests that TND ≥ 90 min is effective in reducing or suppressing fatigue during the night shift, but is not sufficient on its own. The sleep quality at home was associated with recovery from the night shift [70]. Although this study was not able to evaluate sleep other than napping during the night shift, the quality of any sleep, whether main sleep or nap, is important for reducing nurses’ fatigue when both findings are integrated.

The implication for nurses’ occupational health of this study is that napping during night shifts as a countermeasure to fatigue is not only to ensure nap breaks but also for both nurses and managers to recognize the importance of TND for nurses. On 16-h night shifts, the environment should be adjusted to allow for TND ≥ 90 min during every night shift. Intervention studies are also needed to develop policy and programs for nurses’ napping based on evidence.

### 4.3. Limitation

There are several limitations to this study. First, considerable selection bias may be present, including with regard to age and season. The sample size was small because the nurses were restricted to one hospital, and there were differences among the wards. Second, napping was only measured through wearable devices. Sleep depth is not considered. Third, regarding the environment, we could not measure the amount of light to which nurses were exposed, and the noise sources were unclear. In the sound level measurement, possible effects of sound reflection on measured values were not considered, and peak levels of noise were not analyzed. We could not confirm exactly whether nurses used electronic devices before napping. The content and luminance of them were also unclear. Fourth, the effects of 90 min napping were limited to fatigue, and the effects on other outcomes such as performance and accidents are not known. Fifth, only five nurses were able to nap for at least 90 min on all night shifts. Hence, the characteristics of nurses such as, behavioral factors related to napping and fatigue have not been fully verified. For these reasons, it is not possible to generalize our results to all shift workers. In the future, further research is needed on variables that were not covered in this study, such as age, years of nursing experience, sleep depth, and light exposure among large samples. Moreover, it is necessary to verify the effects of nighttime napping on other outcomes.

## 5. Conclusions

The results suggested that nurses experienced less drowsiness after nap breaks, and less fatigue at the end of night shift if they took a nap of at least 90 min when working 16-h night shifts. In addition, the present results suggested that environmental factors such as noise, time spent on electronic devices during nap breaks, and nap break duration were the factors associated with taking a nap of at least 90 min. However, only five nurses took naps for at least 90 min on all four night shifts. The results showed the difficulty of nurses in sleeping for long period during working hours, even at night. To achieve this duration of napping, both individual nurses and nurses’ managers should consider countermeasures for the abovementioned factors.

## Figures and Tables

**Table 1:** Participants’ basic attributes (*n* = 49).

Variables	Mean (*SD*)
Age (years)	28.8 (4.2)
Nursing experience (years)	7.0 (4.2)
Nursing experience in the current ward (years)	3.4 (2.2)
	***n* (%)**
Sex	
Male	3 (6.1)
Female	46 (93.9)
Marital status (Yes)	8 (16.3)
Child-rearing (Yes)	5 (10.2)
Ward	
A (medical)	12 (24.5)
B (medical)	13 (26.5)
C (medical)	5 (10.2)
D (surgical)	5 (10.2)
E (medical)	4 (8.2)
F (surgical)	10 (20.4)

**Notes**. The sample units in this table are persons. **Abbreviations**. SD, standard deviation.

**Table 2:** Nurses’ characteristics (*n* = 49).

Variables	Mean (*SD*)
Sleep	
Subjective mean daily sleep duration (hour)	6.3 (0.9)
	***n* (%)**
Take napping before night shift (Yes)	13 (26.5)
Chronotype	
Definite morning type	0 (0.0)
Moderate morning type	6 (12.2)
Intermediate type	34 (69.4)
Moderate evening type	8 (16.3)
Definite evening type	1 (2.1)
Sleep-related habits	
Frequency of caffeine intake	
Rarely drink (Can’t drink)	18 (36.7)
<2 times/week	10 (20.4)
3–4 times/week	6 (12.2)
5–6 times/week	4 (8.2)
Every day	11 (22.5)
Daily time spent on electronic devices	
30–59 min	1 (2.1)
1–2 h	18 (36.7)
≥2 h	30 (61.2)
Time spent on electronic devices before bedtime	
None	1 (2.1)
<15 min	7 (14.3)
15–29 min	8 (16.3)
30–44 min	17 (34.7)
45–59 min	9 (18.3)
≥60 min	7 (14.3)

**Notes.** The sample units in this table are persons. **Abbreviations.** SD, standard deviation.

**Table 3:** Comparison of nurses’ characteristics based on the number of night shifts with TND ≥ 90 min during four night shifts (*n* = 49).

Variables	None	One/Two Night Shifts	Three/Four Night Shifts	*p*	Post Hoc
	(*n* = 19)	(*n* = 23)	(*n* = 7)		
	**Median (IQR)**				
Age (years)	29.0 (26.0, 30.0)	27.0 (24.0, 31.0)	33.0 (28.0, 33.0)	0.097	
Nursing experience (years)	6.2 (3.3, 8.3)	5.3 (3.2, 9.0)	10.2 (7.2, 12.2)	0.038	None < Three/Four *
Nursing experience in the current ward (years)	2.7 (1.3, 3.8)	3.3 (2.2, 5.3)	4.2 (2.2, 5.0)	0.497	
Subjective mean daily sleep duration (hour)	6.0 (5.5, 7.0)	6.0 (5.5, 7.0)	6.0 (6.0, 7.5)	0.964	
	***n* (%)**				
Gender					
Male	1 (5.3)	2 (8.7)	0 (0.0)	0.999	
Female	18 (94.7)	21 (91.3)	7 (100.0)		
Marital status					
Yes	5 (26.3)	3 (13.0)	0 (0.0)	0.372	
No	14 (73.7)	20 (87.0)	7 (100.0)		
Child-rearing					
Yes	3 (15.8)	2 (8.7)	0 (0.0)	0.682	
No	16 (84.2)	21 (91.3)	7 (100.0)		
Take napping before night shift (Yes)					
Yes	3 (15.8)	7 (30.4)	3 (42.9)	0.302	
No	16 (84.2)	16 (69.6)	4 (57.1)		
Chronotype					
Definite morning type	0 (0.0)	0 (0.0)	0 (0.0)	0.370	
Moderate morning type	2 (10.5)	3 (13.0)	1 (14.3)		
Intermediate type	14 (73.7)	17 (73.9)	3 (42.9)		
Moderate evening type	3 (15.8)	2 (8.7)	3 (42.9)		
Definite evening type	0 (0.0)	1 (4.4)	0 (0.0)		
Frequency of caffeine intake					
Rarely drink (Can’t drink)	5 (26.3)	9 (39.1)	4 (57.1)	0.328	
<2 times/week	3 (15.8)	7 (30.4)	0 (0.0)		
3–4 times/week	3 (15.8)	2 (8.7)	1 (14.3)		
5–6 times/week	3 (15.8)	0 (0.0)	1 (14.3)		
Every day	5 (26.3)	5 (21.8)	1 (14.3)		
Daily time spent on electronic devices					
30–59 min	0 (0.0)	1 (4.4)	0 (0.0)	0.999	
1–2 h	7 (36.8)	8 (34.8)	3 (42.9)		
≥2 h	12 (63.2)	14 (60.8)	4 (57.1)		
Time spent on electronic devices before bedtime					
None	0 (0.0)	1 (4.4)	0 (0.0)	0.721	
<15 min	2 (10.5)	3 (13.0)	2 (28.6)		
15–29 min	4 (21.1)	2 (8.7)	2 (28.6)		
30–44 min	6 (31.6)	10 (43.5)	1 (14.3)		
45–59 min	5 (26.3)	3 (13.0)	1 (14.3)		
≥60 min	2 (10.5)	4 (17.4)	1 (14.3)		

**Notes:** The sample units in this table are persons. *p* values were calculated using Kruskal-Wallis test for continuous variables, while Fisher’s exact test for categorical variables. Post hoc test was conducted using Steel-Dwass test. **Abbreviations.** IQR, interquartile range; SD, standard deviation; TND, total nap duration. * *p* < 0.05.

**Table 4:** Comparison of sleep and napping-related parameters between TND ≥ 90 min and TND < 90 min groups (*n* = 196).

Variables	TND < 90 min (*n* = 137)	TND ≥ 90 min (*n* = 59)	*t*	*p*
Sleep or Nap before the night shifts				
Last awakening time (h:m)	10:52 (2:10)	11:39 (1:57)	2.42	0.016
Last sleep or nap duration (hour)	7.3 (3.2)	6.8 (3.6)	−0.93	0.355
Awakening duration until nap breaks (hour)	13.9 (3.0)	13.5 (2.5)	−0.76	0.450
Nap breaks and napping during night shifts				
Start time of nap breaks (h:m)	0:45 (1:53)	1:12 (1:58)	1.53	0.128
TIB (min)	104.6 (27.7)	160.2 (32.3)	12.24	<0.001
Start time of lying (h:m)	1:02 (1:53)	1:23 (1:57)	1.19	0.235
Start time of napping (h:m)	1:30 (1:50) ^a^	1:40 (1:54)	0.58	0.563
SL (min)	23.2 (18.9) ^a^	17.2 (12.3)	−2.23	0.027
TND (min)	60.5 (20.4)	120.9 (27.1)	17.15	<0.001
SE (%)	59.6 (18.2)	75.9 (9.7)	6.48	<0.001
WASO (min)	11.8 (13.6) ^a^	16.3 (14.4)	2.07	0.040
Frequency of awakenings during napping (Times)	1.7 (1.4) ^a^	2.9 (1.8)	5.04	<0.001
Frequency of postural changes (Times)	2.5 (2.4) ^a^	4.3 (2.9)	4.32	<0.001
End time of napping (h:m)	2:44 (1:46) ^a^	3:58 (1:50)	4.36	<0.001
BOT (min)	6.4 (4.6) ^a^	5.9 (2.8)	−0.80	0.426
End time of lying (h:m)	2:47 (1:48)	4:03 (1:50)	4.54	<0.001
End time of nap breaks (h:m)	2:58 (1:46)	4:13 (1:50)	4.45	<0.001
Subjective evaluation (%)	47.1 (21.5) ^b^	65.9 (22.0)	5.46	<0.001

**Notes.** The sample units in this table are the number of night shifts. The data are presented as mean (standard deviation). *p* values were calculated using the student’s *t*-test. There were four night shifts which were not as determined getting sleep. 17 night shifts of subjective evaluation were excluded because they were not completed. **Abbreviations.** BOT, bed-out time; TIB, time in bed; TND, total nap duration; SE, sleep efficiency; SL, sleep latency; WASO, wake after sleep onset. ^a^ *n* = 133. ^b^ *n* = 120.

**Table 5:** Environment during night shifts between TND ≥ 90 min and TND < 90 min groups (*n* = 196).

Variables	Mean (*SD*) or *n* (%)		*t*	*p*
	TND < 90 min (*n* = 137)	TND ≥ 90 min (*n* = 59)		
Napping environment				
Illuminance (lux)	18.6 (89.3)	11.0 (58.5) ^a^	−0.59	0.556
Noise level (dB *L*_Aeq, napping_)	42.3 (4.7)	39.3 (4.8)	−4.11	<0.001
Temperature (°C)	25.1 (1.3)	24.9 (1.0) ^a^	−1.05	0.294
Humidity (%)	66.2 (7.5)	64.3 (6.3) ^a^	−1.70	0.091
Unpleasant smells (Yes)	2 (1.5)	0 (0.0)		0.999
Japanese futons (Yes)	40 (29.2)	31 (52.5)		0.002
Folding beds (Yes)	36 (26.3)	1 (1.7)		<0.001
Beds in vacant hospital room (Yes)	23 (16.8)	14 (23.7)		0.320
Sofa beds (Yes)	14 (10.2)	7 (11.9)		0.802
Stretchers (Yes)	20 (14.6)	0 (0.0)		0.001
Examination tables (Yes)	4 (2.9)	6 (10.2)		0.069
Bed sheets (Yes)	124 (90.5)	54 (91.5)		0.999
Quilt (Yes)	125 (91.2)	55 (93.2)		0.781
Toweling blankets (Yes)	9 (6.6)	10 (17.0)		0.034
Pillow (Yes)	117 (85.4)	52 (88.1)		0.822
Ways of spending breaks				
Caffeine consumption before nap breaks (Yes)	67 (48.9)	23 (40.4) ^b^		0.343
Carrying a PHS with the nurse call system (Yes)	8 (5.8)	10 (17.0)		0.028
Time spent on electronic devices (min)	29.8 (26.8)	23.6 (26.5)	−1.50	0.136
Changing clothes (Yes)	8 (5.8)	3 (5.1)		0.999
Using a sleep mask (Yes)	6 (4.4)	4 (6.8)		0.492
Using earplugs (Yes)	2 (1.5)	0 (0.0)		0.999
Watching television (Yes)	4 (2.9)	4 (6.8)		0.245
Listening to music (Yes)	9 (6.6)	1 (1.7)		0.287
Doing remaining work (Yes)	17 (12.4)	3 (5.1)		0.196
Eating (Yes)	27 (19.7)	21 (35.6)		0.029
Working Environment				
Number of nurses	3.0 (0.6)	3.1 (0.7)	0.14	0.886
Number of care workers	0.2 (0.4)	0.4 (0.5)	1.54	0.125
Number of hospitalized patients	22.1 (4.3)	21.0 (4.1)	−1.68	0.095
Number of patients each nurse responsible for	8.2 (4.4)	7.4 (3.0)	−1.25	0.213
Supper breaks (Yes)	137 (100.0)	59 (100.0)		0.999
Nap break duration (min)	138.3 (27.7)	182.2 (39.6)	8.88	<0.001
Steps	10,367.2 (2652.9) ^c^	8918.6 (2199.9) ^d^	−3.23	0.002
Steps before napping per hour	736.5 (204.3) ^c^	648.6 (193.7) ^d^	−2.47	0.014
Steps after napping per hour	752.7 (229.7) ^e^	769.5 (238.6) ^b^	0.46	0.648

**Notes.** The sample units in this table are the number of night shifts. *p* values were calculated using student’s *t*-tests for continuous variables, while Fisher’s exact tests were used for categorical variables. Equipment bugs and incomplete questionnaires were excluded. **Abbreviations.** dB *L*_Aeq, napping_, decibel equivalent A-weighted sound pressure level during napping; PHS, personal handy-phone system; TND, total nap duration. ^a^ *n* = 58. ^b^ *n* = 57. ^c^ *n* = 129. ^d^ *n* = 43. ^e^ *n* = 136.

**Table 6:** Environmental factors associated with night shift of TND ≥ 90 min (*n* = 172).

Variables	a*OR* [95% CIs]	*p*
Napping Environment		
Noise level (dB *L*_Aeq, napping_)	0.88 [0.78, 0.98]	0.022
Humidity (%)	0.94 [0.87, 1.02]	0.152
Japanese futons		
Yes	1.19 [0.34, 4.14]	0.787
No	1.00 [Ref]	
Folding beds		
Yes	0.73 [0.06, 9.48]	0.807
No	1.00 [Ref]	
Examination tables		
Yes	15.02 [0.80, 283.19]	0.071
No	1.00 [Ref]	
Toweling blankets		
Yes	1.44 [0.29, 7.07]	0.655
No	1.00 [Ref]	
Ways of spending breaks		
Carrying a PHS with nurse call system		
Yes	1.71 [0.13, 22.86]	0.686
No	1.00 [Ref]	
Time spent on electronic devices (min)	0.97 [0.94, 0.99]	0.009
Doing remaining work		
Yes	0.24 [0.03, 2.01]	0.188
No	1.00 [Ref]	
Eating		
Yes	1.78 [0.57, 5.54]	0.319
No	1.00 [Ref]	
Working Environment		
Number of care workers (person)	1.17 [0.22, 6.30]	0.854
Number of hospitalized patients (person)	0.87 [0.74, 1.04]	0.124
Nap break duration (min)	1.04 [1.02, 1.07]	<0.001
Steps before napping (100 steps/hour)	0.75 [0.55, 1.03]	0.078

**Notes.** Multivariable logistic regression analysis with awaking duration until nap break set was as a covariate. The sample units in this table are the number of night shifts. The 24 night shifts were excluded because the wearable devices could not record it due to a bugs. **Abbreviations.** CI, confidence interval; dB *L*_Aeq, napping_, decibel equivalent A-weighted sound pressure level during napping; a*OR*, adjusted odds ratio; PHS, personal handy-phone system; TND, total nap duration.

## Data Availability

Due to the nature of this research, nurses participating in this study did not agree for their data to be shared publicly, so data sharing is not applicable.

## Supplementary Materials

The following supporting information can be downloaded at: https://www.mdpi.com/article/10.3390/ijerph19159429/s1, Figure S1: Comparison of the mean complaint rate (%) of the Cumulative Fatigue Symptoms Index by number of naps with TND ≥ 90 min during four night shifts; Figure S2. Correlation between indicators of body movement and noise level during napping; Figure S3. Correlation of TIB and TND with change in fatigue before and after the nap breaks.
