# Supplementary material for: Effects of 90 Min Napping on Fatigue and Associated Environmental Factors among Nurses Working Long Night Shifts: A Longitudinal Observational Study
Source: Int J Environ Res Public Health. 2022 Aug 1;19(15):9429. doi: 10.3390/ijerph19159429 (PMC9367999; doi:10.3390/ijerph19159429)
Supplement: Supplementary file 1 [file ijerph-19-09429-s001.zip › ijerph-1780481-supplementary.pdf]

International Journal of
Environmental Research and Public Health

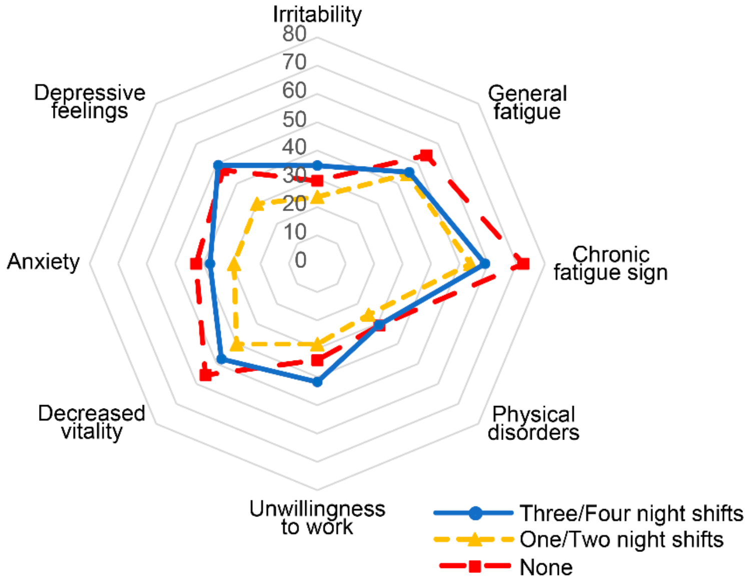

Figure S1. Comparison of the mean complaint rate (%) of the Cumulative Fatigue Symptoms Index by number of naps with TND ≥ 90 min during four night shifts

Notes. The sample units in this figure are persons. The radar chart indicates the mean complaint rates of the Cumulative Fatigue Symptoms Index. The mean complaint rates for each category were compared by the number of naps with TND > 90 min during four night shifts among nurses. Blue line: Three/Four night shifts group (n = 7, circles), Yellow line: One/Two night shifts group (n = 23, triangles), Red line: None group (n = 19, squares), respectively. No significant differences were shown in each category. **Abbreviations.** TND, total nap duration.

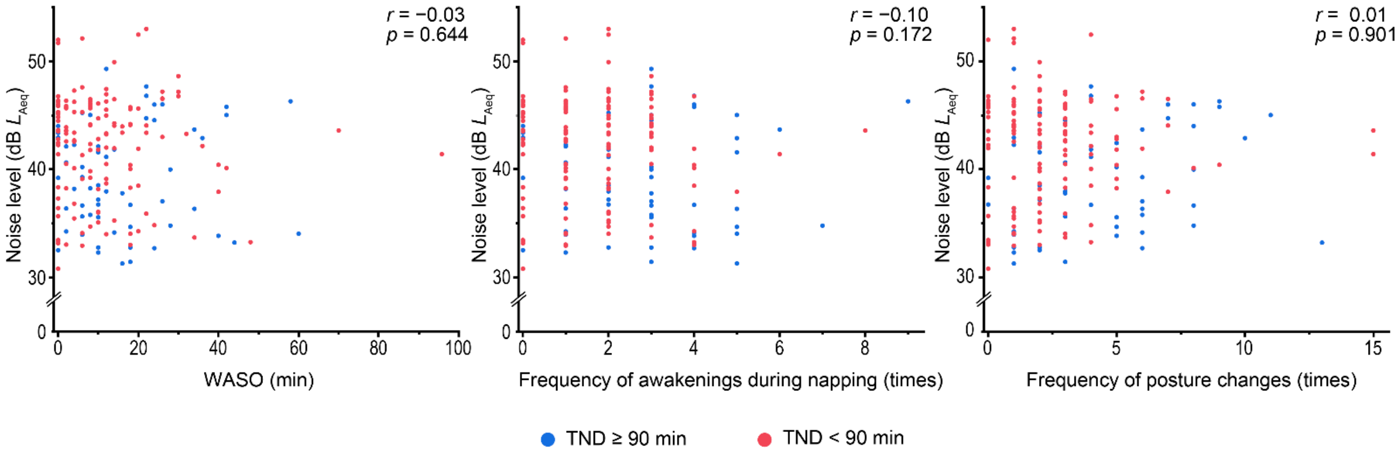

Figure S2. Correlation between indicators of body movement and noise level during napping

Notes. The sample units in this figure are night shifts. The vertical axis indicates the noise level, equivalent A-weighted sound pressure level, during napping calculated by the digital sound level meter with a data logger. The horizontal axis indicates the indicators of body movement during napping calculated by the wearable device. The blue dots are TND ≥ 90 min group, while the red dots are TND < 90 min group. Pearson's r correlation coefficient was analyzed between the indicators of body movement and the noise level. **Abbreviations.** dB L_{Aeq}, decibel equivalent A-weighted sound pressure level; TND, total nap duration.

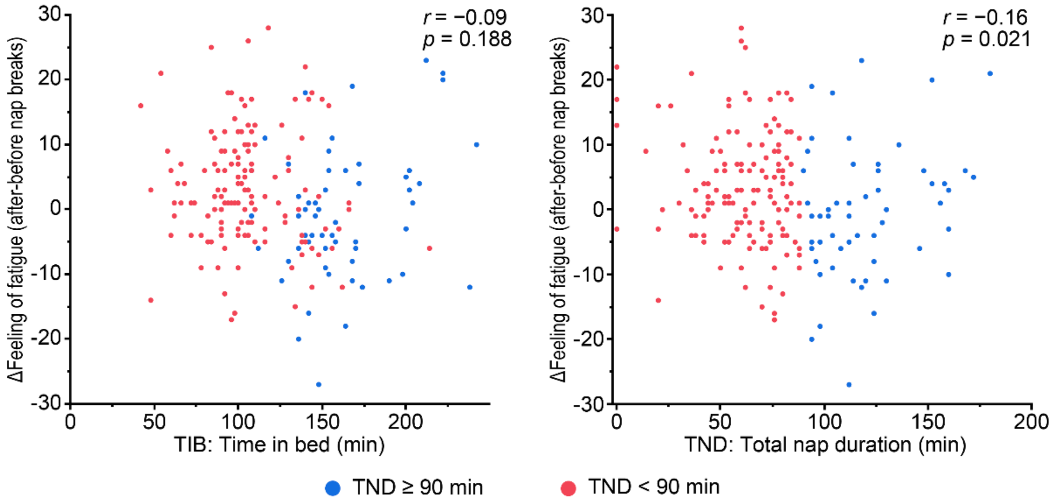

Figure S3. Correlation of TIB and TND with change in fatigue before and after the nap breaks

Notes. The sample units in this figure are night shifts. The vertical axis indicates the change in fatigue before and after the napping. The change in fatigue was calculated by subtracting the total score for feeling of fatigue (*Jikaku-sho shirabe*) before the nap break from after the nap break. The horizontal axis indicates nap-related variables calculated by the wearable device. The blue dots are TND ≥ 90 min group, while the red dots are TND < 90 min group. Pearson's *r* correlation coefficient was analyzed between the nap-related variables and the change in feeling of fatigue. **Abbreviations.** TIB, time in bed; TND, total nap duration.
